# Membrane Remodeling Driven by Shallow Helix Insertions via a Cooperative Mechanism

**DOI:** 10.3390/membranes15040101

**Published:** 2025-04-01

**Authors:** Jie Hu, Yiben Fu

**Affiliations:** 1 Key Laboratory of Biomedical Materials and Engineering of the Ministry of Education, South China University of Technology, Guangzhou 510006, China; 2 National Engineering Research Center for Tissue Restoration and Reconstruction, South China University of Technology, Guangzhou 510006, China; 3 School of Biomedical Sciences and Engineering, South China University of Technology, Guangzhou International Campus, Guangzhou 511442, China; 4 Guangdong Provincial Key Laboratory of Biomedical Engineering, South China University of Technology, Guangzhou 510006, China

**Keywords:** membrane deformation, membrane model, helix insertion, membrane-mediated interactions

## Abstract

Helix-membrane interactions are key to membrane deformation and play significant biological roles. However, systematic studies on the mechanisms behind these interactions are limited. This study uses a continuum membrane model to investigate how shallowly inserted helices interact with biological membranes, focusing on membrane deformation and the cooperative effects of multiple helices. Our findings show that even short helices (2 nm in length) can induce anisotropic membrane deformation. Longer helices and deeper insertions result in more significant deformations, and the spatial arrangement of helices affects the nature of these deformations. The perturbation area (PA) and perturbation extent (PE) are quantified to describe membrane deformation, revealing stronger cooperative effects in parallel insertions and more complex deformations in other arrangements. Additionally, membrane properties, such as lipid composition, influence the extent of deformation. In multi-helix systems, we observe local clustering behavior when perturbations are strong enough, with cooperativity varying based on helix length, insertion depth, and membrane composition. This study provides criteria for helix cooperativity, advancing our understanding of helix–membrane interactions and their biological significance in processes like membrane remodeling.

## 1. Introduction

Protein binding to cytoplasmic membranes plays a critical role in various biological processes, including endocytosis [1,2], signaling [3], and cell division [4]. To stabilize their membrane interactions, proteins employ multiple mechanisms, one of which involves the insertion of an amphipathic peptide or terminal α-helix into the membrane. Examples of this include the epsin N-terminal homology (ENTH) domain [5], bin-amphiphysin-rvs (BAR) proteins [6], and septins [7]. It has been observed that many α-helices can induce membrane curvature by deforming the membrane’s geometric shape, even when they are only shallowly inserted [8]. Beyond inducing curvature, such α-helices/peptides have also demonstrated curvature-sensing capabilities [9], which have garnered significant attention from researchers across biology, physics, and engineering disciplines [10,11,12,13,14].

The behavior of helix insertion into membranes has been extensively studied through both experimental and computational approaches. Experiments have shown that some amphipathic helices can insert into membranes and exhibit higher affinity for highly curved membranes [15,16,17]. Similarly, BAR domain proteins have been observed to insert their α-helices into membranes, aligning with one another [18]. On the computational side, molecular dynamics simulations have provided insight into the mechanisms of helix insertions [19], revealing that this process alters the distribution of nearby lipids and reduces lipid-packing defects [20,21,22]. Furthermore, solid membrane models based on elasticity theory have successfully captured the stress distribution surrounding a single helix insertion [23], and subsequent studies have confirmed that such insertion events can sense membrane stress [24].

While the insertion of a single helix into a membrane provides a simplified framework for studying helix–membrane interactions, the scenario involving two helices has become a prototypical system for exploring the mechanical cooperation among membrane-bound helices. It has been shown that transmembrane proteins or particles can exhibit membrane-mediated interactions due to their large-scale perturbation of the membrane [25,26]. Interestingly, shallowly inserted helices can also interact indirectly through the membrane [27], even though the membrane deformations they induce are weaker than those caused by transmembrane proteins. Theoretical analyses and simulations have elucidated how two parallel helices interact mechanically via the membrane deformation they induce [28,29]. However, several open questions remain, such as how multiple helices behave collectively on the membrane surface and whether the orientation of helices and membrane composition influence distribution.

In this study, we investigate α-helices binding to membranes with shallow insertion depths. Our computational simulations utilize a recently developed continuum membrane model that captures the mechanics of the bilayer membrane’s two leaflets [30]. First, we quantitatively examine how a single helix perturbs the membrane by varying the helix length and insertion depth. We then explore how the number and orientation of helices influence membrane structure, which, in turn, regulates the distribution of the helices on the membrane. Our findings demonstrate that α-helices can exhibit mechanical cooperation through the induced membrane deformation, and this cooperation is governed by the physical properties of both the helices and the membrane.

## 2. Methods

### 2.1. Continuum Membrane Model

To comprehensively demonstrate how α-helix insertion perturbs the bilayer membrane, we conducted computational simulations using our recently developed continuum membrane model, which has been validated against molecular dynamics simulations and experimental results [30]. Below, we summarize the key elements of the model.

The bilayer membrane is represented by three layers of triangular meshes based on finite element analysis. The top layer of the mesh represents the neutral surface of the top leaflet (monolayer) of the bilayer membrane. This neutral surface is approximately located at the interface between the lipid polar heads and hydrophobic tails, where monolayer bending elasticity and area elasticity are decoupled [23,31,32]. Similarly, the bottom layer of the mesh represents the opposite monolayer, while the middle layer corresponds to the interface between the two monolayers. The monolayer energetics include three main terms: curvature energy (modeled using the Helfrich-Canham-Evans model [33,34,35]), area energy [36,37], and height/thickness energy (transformed equivalently from tilt energy [38]). The total energy of a monolayer is expressed as follows:

(1) $$E_{mono}=\int \left[\frac{1}{2}\kappa_{c}{\left(2c-c_{0}\right)}^{2}+\frac{1}{2}\kappa_{h}{\left(3{\rho_{h}}^{2}-2\rho_{h}\right)}^{2}\right]dA+\frac{1}{2}\mu_{A}\frac{{\left(A-A_{0}\right)}^{2}}{A_{0}},$$

where $\kappa_{c}$ is the monolayer bending modulus, 2c is the surface curvature and $c_{0}$ is the spontaneous curvature of the monolayer. The parameter $\kappa_{h}$ is the height elasticity constant that equals three times the tilt modulus, and $\rho_{h}$ is the height strain capturing the monolayer thinning. The integral of the curvature energy and height energy covers the whole area of the monolayer surface A, with $A_{0}$ as the equilibrium area and $\mu_{A}$ as the elastic constant. The bilayer membrane energy is computed as the sum of the two monolayers’ energetics. For simplicity, we assume a symmetric bilayer membrane where the two monolayers share identical parameters.

The α-helix insertion is modeled by selecting specific regions (triangles) of the mesh as insertion sites and assigning unique parameter values to represent the insertion, which is based on the general principles of protein-membrane interactions [39]. The key modifications include: (1) Spontaneous curvature: Insertion regions are assigned a spontaneous curvature, $c_{0,ins}$, typically larger than 0 (e.g., $c_{0,ins}=0.3$ nm^−1^ according to the previous study [11]). (2) Equilibrium height reduction: The equilibrium height at the insertion zone is decreased by $∆h_{0}$ due to lipid deformation or wrapping around the helix. Larger $∆h_{0}$ values correspond to deeper insertions or greater deformation of lipids. Based on prior studies, $∆h_{0}$ typically ranges from 0.1 to 0.3 nm. (3) Helix area: The helix occupies a specific area ($a_{0}$) on the membrane, increasing the equilibrium area of the monolayer by $a_{0}$.

Using this continuum membrane model, we simulate one or more helix insertions into one side of the bilayer membrane (the outer layer). By minimizing the system’s energy, we obtain the equilibrium membrane structure and corresponding energy. Notably, our continuum membrane model employs static numerical calculations for given parameters, rather than capturing dynamic or stochastic processes, allowing us to focus solely on the final equilibrium state of the simulations.

### 2.2. Quantitative Description of Membrane Deformation

Helix insertion perturbs the membrane, leading to localized deformation around the insertion site. This deformation is typically limited to a few nanometers from the helix center. Changes in helix properties, such as length or insertion depth, directly affect the extent of membrane deformation, shown by our results below. For an initially flat membrane in the *x*-*y* plane, deformation can be characterized by two key features: deformation area in the *x*-*y* plane and local displacement in the *z*-direction, such as arching and sinking. To describe these features quantitatively, we introduce two indices:

#### 2.2.1. Perturbation Area (PA)

The perturbation area (PA) quantifies the extent of local deformation around the helix. Using finite element analysis [36], we calculate the displacement (∆z) of each triangular element’s center along the *z*-axis. PA is defined as the sum of the areas of all triangles where $\left|∆z\right|>\varepsilon$, with ε being a small threshold (e.g., ε = 0.01 nm). Formally,

(2) $$PA=\sum_{i}\left\{a_{i}|\varepsilon <\left|∆z_{i}\right|\right\},$$

where $a_{i}$ is the area of the *i*-th triangle, and $∆z_{i}$ is the *z*-displacement of its center. PA is expressed in units of area (nm^2^), with larger PA values indicating broader deformation caused by the helix.

#### 2.2.2. Perturbation Extent (PE)

The perturbation extent (PE) describes the magnitude of membrane deformation in the *z*-direction, capturing the degree of arching or sinking. PE is defined as follows:

(3) $$PE=\sqrt{\frac{\sum_{i}{\left(∆z_{i}\cdot a_{i}\right)}^{2}}{\sum_{i}a_{i}}},$$

where $a_{i}$ and $∆z_{i}$ are defined as above. PE is also expressed in units of area (nm^2^). Higher PE values indicate more pronounced deformation, where the membrane arches upward or sinks downward.

## 3. Results

### 3.1. Membrane Deformation Induced by a Single Helix

To systematically investigate the mechanism behind helix-induced membrane deformation and assess its generality, we conducted computational simulations using the bilayer continuum membrane model. The membrane properties were based on DOPC lipid membranes by default (Table 1). In these simulations, the helix was assumed to be inserted exclusively on the outer layer of the membrane. We standardized the helix width to 1.0 nm, based on the structural size of membrane curvature-sensing and -inducing helices; for example, the amphiphilic helix in the N-terminal of the ENTH domain is about 1.0 nm wide and 2.0 nm long [5]. To examine the effects of helix length and insertion depth, we systematically varied the parameters *L* and $∆h_{0}$, respectively. The spontaneous curvature of the helix was fixed at $c_{0,ins}$ = 0.3 nm^−1^.

#### 3.1.1. Radial Perturbation Induced by Helix Insertion

**Figure 1:**
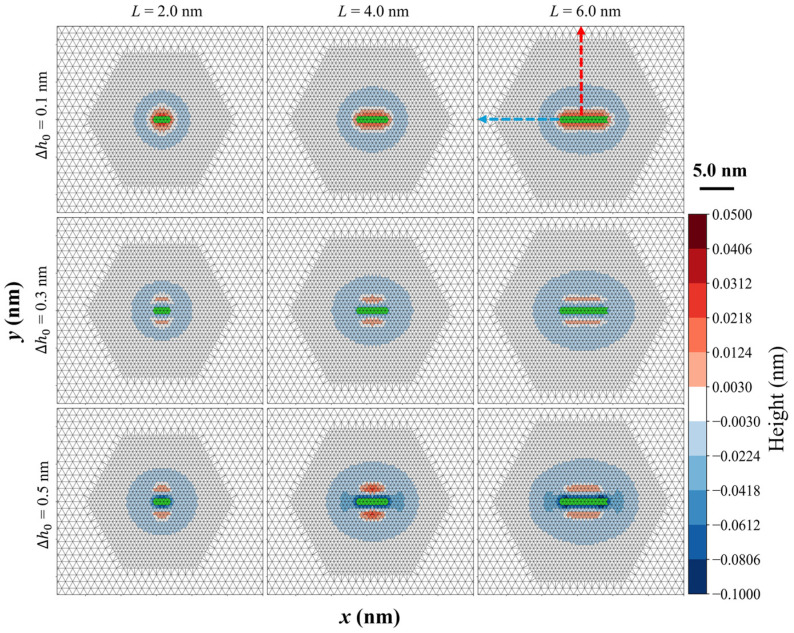
The membrane deformation induced by a single helix with varying lengths and insertion depths. The matrix displays deformation patterns across three insertion depths and three helix lengths. The three columns, from left to right, correspond to helix lengths *L* = 2, 4, and 6 nm, respectively. The three rows, from top to bottom, represent $∆h_{0}$ = 0.1, 0.3, and 0.5 nm, respectively. Color coding depicts membrane thickness or height variations: red indicates increased thickness (elevation or arching), blue represents show decreased thickness (thinning or sink), and green marks the helix insertion site.

The deformation of the membrane caused by a single helix is illustrated in Figure 1. The results show that the membrane perturbation displaces a radial distribution centered around the helix insertion site. For a constant $∆h_{0}$, the deformation region in the outer membrane layer expands as the helix length increases. For shorter helices, the deformation zone is approximately circular. Figure S1 illustrates the circular deformation of the membrane caused by a single assumed small helix. However, for longer helices, the perturbation zone adopts an elliptical shape, with the major axis aligned along the length of the helix. The membrane deformation is most pronounced near the helix insertion site, gradually decaying several nanometers away from the helix center. It is also observed that variations in the insertion depth alter the membrane perturbation, particularly affecting the membrane height adjacent to the helix. The inner membrane layer exhibits similar deformation patterns to the outer layer (Figure S2), suggesting that insertion of the helix into one leaflet induces deformation in the opposite leaflet. In the continuum membrane model to simulate the helix–membrane system, the two leaflets are assumed to be tightly coupled, preventing the formation of vacuum-like isolation zones between them [30], such that both relax simultaneously to reach an equilibrium membrane structure.

#### 3.1.2. Anisotropic Perturbation Induced by Helix Insertion

**Figure 2:**
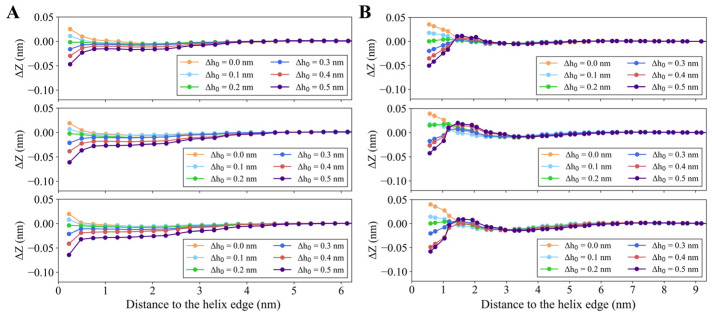
Directional analysis of membrane height deformation around the helix insertion. (**A**) Height profiles along the *x*-axis, captured along the blue dashed line in Figure 1. (**B**) Height profiles along the *y*-axis, captured along the red dashed line in Figure 1. The three rows are shown for different helix lengths, from top to bottom, *L* = 2, 4, and 6 nm, respectively.

Further analysis reveals that, for a fixed helix length, both the spatial distribution and the magnitude of membrane deformation depend on the insertion depth, as shown in Figure 2. When $∆h_{0}$ ≤ 0.1 nm, the membrane near the insertion site remains arched, indicating that the helix’s spontaneous curvature ($c_{0,ins}$) dominates, as $c_{0,ins}$ is a positive value that elevates the membrane outward. However, as the insertion depth increases, distinct membrane remodeling patterns are triggered. Larger $∆h_{0}$ values cause the membrane to sink near helix insertion. Notably, membrane deformation along the x- and *y*-axis is different. Figure 2A illustrates that the membrane height along the *x*-axis is influenced by $∆h_{0}$. Specifically, for a 2.0 nm helix with $∆h_{0}$ = 0.2 nm, the deformation extends approximately 1.0 nm along the *x*-axis, whereas this range increases beyond 2.0 nm when $∆h_{0}$ is increased to 0.4 nm. Figure 2B shows membrane height variations along the *y*-axis, which differ from the *x*-axis observations, particularly when $∆h_{0}$ > 0.2 nm. For instance, when $∆h_{0}$ = 0.4 nm, the membrane height remains unaffected far from the helix edge, rises about 2.0 nm near the helix edge, and then decreases closer to the helix. This indicates that membrane deformation is more pronounced along the *y*-axis than along the *x*-axis. This anisotropic deformation can be attributed to the asymmetric geometry of the helix, where its width (1.0 nm) is smaller than its length. In comparison, the symmetric insertion induces isotropic membrane deformation (Figure S1). As a result, the helix binding direction fundamentally determines the anisotropic nature of membrane deformation.

Analysis of the inner membrane layer shows a similar anisotropic deformation pattern (Figure S2), although the deformation process is more straightforward compared to the outer layer. Since the helix is inserted only in the outer layer of the membrane, with no insertion in the inner layer, the outer membrane lipids experience more complex deformation, especially near the helix, than the inner layer lipids.

#### 3.1.3. Quantitative Analysis of Membrane Deformation

**Figure 3:**
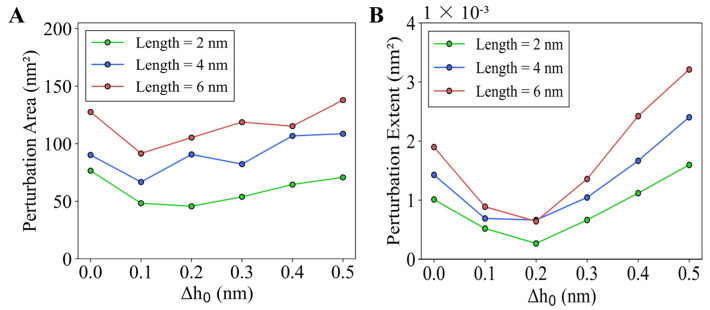
Effects of the helix length and insertion depth on (**A**) perturbation area and (**B**) perturbation extent of the outer layer of the membrane induced by a single helix.

To quantify the membrane deformation, we analyzed both the perturbation area (PA) and perturbation extent (PE). As shown in Figure 3A, the PA value increases with the helix length, consistent with our previous observations. For instance, at $∆h_{0}$ = 0.2 nm, increasing the helix length from 2.0 nm to 6.0 nm results in a 130.61% increase in PA, from 45.66 nm^2^ to 105.29 nm^2^. While varying insertion depth with a fixed helix length also influences PA, this effect is less pronounced than the impact of helix length. Figure 3B demonstrates the dependence of PE on both insertion length and insertion depth. Larger insertion lengths lead to higher PE values. When $∆h_{0}$ is increased from 0 to 0.4 nm, PE first decreases and then increases, reaching a minimum at $∆h_{0}$ = 0.2 nm. This can be explained by the opposing effects of the two key parameters, $∆h_{0}$ and $c_{0,ins}$, on the perturbation of the outer membrane layer. While $c_{0,ins}$ tends to elevate the membrane surface, $∆h_{0}$ causes it to sink. When $c_{0,ins}$ = 0.3 nm^−1^ and $∆h_{0}$ = 0.2 nm, these opposing effects balance, resulting in a nearly flat membrane surface (Figure 2) and minimizing the perturbation extent PE.

Similarly, we calculated PA and PE for the inner membrane layer (Figure S2). The PA of the inner layer is almost identical to that of the outer layer; however, the PE of the inner layer differs. Specifically, PE of the inner layer increases with $∆h_{0}$, giving a minimum near $∆h_{0}$ = 0 nm. This suggests that the deformation of the inner layer is not subject to the competition between $∆h_{0}$ and $c_{0,ins}$, as the helix is inserted only in the outer layer.

These findings indicate that helix-induced membrane deformation is a synergistic function of both helix length and insertion depth. Specifically, the perturbation extent is determined by the combined effects of these two parameters, while the perturbation area is primarily determined by the helix length rather than the insertion depth, offering new mechanistic insights into amphiphilic helix-mediated membrane remodeling. Furthermore, the observed influence of helix binding directionality on the anisotropic propagation of membrane deformation provides a valuable basis for investigating membrane responses to multiple helix insertions.

### 3.2. Membrane Deformation Induced by Two Helices

**Figure 4:**
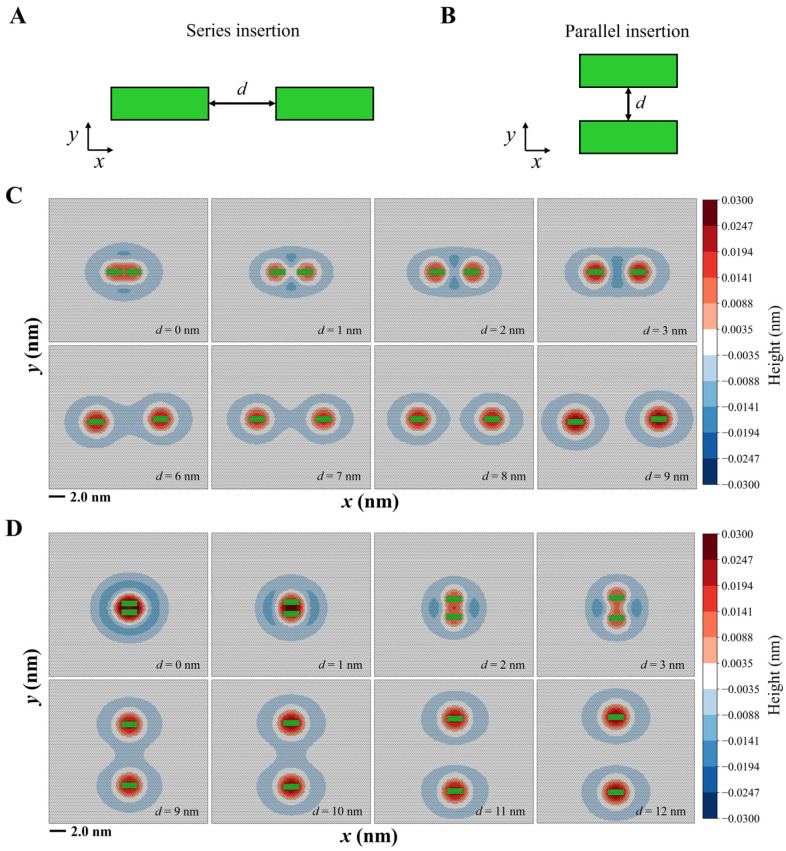
Two helices induce mixed membrane deformations. Schematics of two alignment types: series insertions (**A**) and parallel insertions (**B**). The parameter *d* represents the spacing distance between the two helices. (**C**) Membrane deformation resulting from series insertions. (**D**) Membrane deformation resulting from parallel insertions. Simulations were performed with DOPC membranes, and the helix length is *L* = 2 nm and $∆h_{0}$ = 0.15 nm.

Given the anisotropic feature of helix-induced membrane deformation, we sought to explore the potential coupling effects between two helix insertions on the same side of biological membranes by varying the helix length, insertion depth, and alignments. For simplification, two types of helix alignments were considered in the two-helix system (Figure 4): (1) series insertions, where two helices are positioned end-to-end, aligning their axes along a single line; and (2) parallel insertions, where two helices are positioned parallel to each other with their axes perpendicular to the line connecting their centers.

#### 3.2.1. Two Helices Induce Mixed Membrane Deformation

By fixing the helix length at 2 nm and $∆h_{0}$ at 0.15 nm, we examined how the alignment influences the behavior of the two helices as the inter-helical distance varies. For the series insertions, the deformation zones of the two helices merge when they are close to each other (Figure 4C). As the inter-helical distance increases, the merged area becomes smaller, with complete segregation of deformation zones occurring at approximately 8.0 nm. This suggests a transition from a merged to a segregated deformation pattern in the series insertions. Similarly, parallel insertions also exhibit merge-to-segregation membrane deformation (Figure 4D), with the merged area persisting at an inter-helical distance of 10.0 nm. Only when the inter-helical distance exceeds 10.0 nm does the merged area completely disappear. This is attributed to the anisotropic nature of helix-induced membrane deformation. Since membrane deformation around a helix is more pronounced along the *y*-axis than the *x*-axis (Figure 2), a longer spacing distance is required for the parallel insertions to eliminate the merged area compared to the series insertions.

Helix length and insertion depth also affect membrane deformation. Our results indicate that increasing the insertion depth alters the deformation surrounding each helix. A greater insertion depth leads to a longer segregation distance, whereas smaller insertion depths may result in a similar segregation distance (Figure S3A). The helix length has an even more substantial impact on the mixed membrane deformation, particularly in the case of parallel insertions (Figure S3B). As the helix length increases, the merged area between the two helices becomes larger; thus, a longer inter-helical distance will be required to separate the deformed membrane regions. This is because longer helices induce stronger membrane deformation, both in perturbation area and extent (Figure 3). As a result, two longer parallel insertions exhibit a larger merged area compared to shorter ones, necessitating a longer spacing distance to achieve separation.

Since the inner leaflet of the membrane is also deformed through mechanical interactions with the outer leaflet, our simulations reveal that the inner layer exhibits mixed deformation in response to the two helix insertions on the outer layer (Figure S4). This mixed deformation is modulated by the arrangement of the helices, with different color maps observed for the series and parallel insertions. Notably, the merged area on the inner leaflet vanishes at a similar inter-helical distance as observed in the outer leaflet. This alignment is due to the strong coupling between the two membrane leaflets.

#### 3.2.2. Mixed Membrane Deformation Exhibits a Stepwise Process

**Figure 5:**
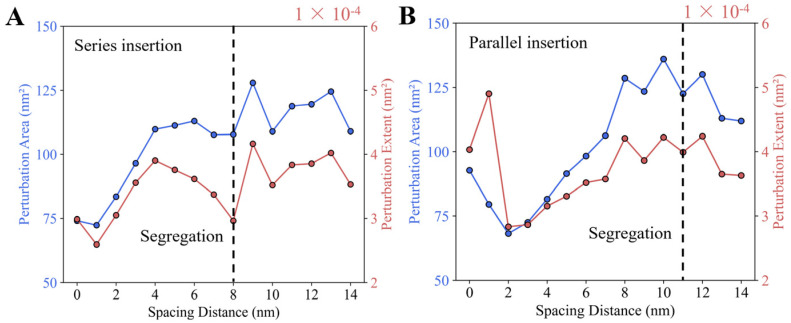
Membrane deformation exhibits a stepwise pattern as the spacing distance changes. Quantitatively, both the perturbation area and perturbation extent vary with spacing distance for the series insertions (**A**) and parallel insertions (**B**). The dashed lines indicate the spacing distance at which the mixed membrane deformation disappears, as shown in Figure 4. In the computational simulations, the insertion depth is $∆h_{0}$ = 0.15 nm and the helix length is *L* = 2 nm.

A quantitative analysis of the membrane deformation in the two-helix system was performed by calculating the perturbation area (PA) and perturbation extent (PE). For series insertions, the PA as a function of inter-helical distance shows a stepwise process. As illustrated in Figure 5A, PA increases as the inter-helical distance *d* increases from 1.0 to 4.0 nm, after which it stabilizes. This suggests a significant change in the coupling effect between the two series insertions around the inter-helical distance of 4.0 nm. A similar trend is observed for PE, with a turning point occurring at a spacing distance of 4.0 nm. This suggests that PA and PE quantitatively capture the coupling effect between the two series insertions. The fact that the turning point of PA and PE occurs before the separation distance highlights a discrepancy between the quantitative and qualitative analyses of membrane deformation. We attribute this to the criteria used in data analysis. For instance, in Figure 4C, at the spacing distance of *d* = 6.0 nm, the height deformation of mixed membrane deformation between the two helix insertions is less than 0.0088 nm. If this height deformation is considered negligible, the separation distance at which mixed membrane deformation disappears would be shorter than 6.0 nm, aligning more closely with the turning point observed in Figure 5A.

For parallel insertions, the coupling effect between the two helices also follows a stepwise pattern dependent on inter-helical distance *d*, as shown in Figure 5B. As the inter-helical distance increases from 2.0 to 8.0 nm, PA increases consistently. Beyond *d* = 8.0 nm, PA reaches a stable value. Similarly, PE exhibits a turning point at *d* = 8.0 nm, indicating a significant shift in the coupling effect at this inter-helical distance. This turning point occurs earlier than the separation distance of 11.0 nm, where mixed membrane deformation disappears, as shown in Figure 4D. As previously explained, this discrepancy arises from differences in the setup of quantitative and qualitative analyses. Additionally, when the parallel insertions are in close proximity (*d* ≤ 1.0 nm), both PA and PE are larger than their values at *d* = 2.0 nm, indicating an extreme coupling effect between the two helices on membrane deformation. However, the scenario where the two helices are fully adhered (*d* = 0 nm) is overly idealized and unlikely to occur in reality, as the amino acid side chains in proteins exhibit volume exclusion. Therefore, in the subsequent analysis, we focus on cases where the minimum inter-helix distance is 0.5 nm.

It is noteworthy that the turning point inter-distance in Figure 5B is larger than that in Figure 5A, indicating that parallel insertions exhibit a longer coupling range than series insertions. As we explained previously, this extended coupling range in parallel insertions is attributed to the anisotropic nature of the membrane deformation induced by each helix. Since each helix induces stronger deformation along the *y*-axis than the *x*-axis, parallel insertions exhibit a more significant coupling effect than series insertions when the inter-helical distances are the same. As a result, parallel insertions require a larger inter-helical distance to diminish the coupling effect compared to series insertions.

#### 3.2.3. Parallel Insertions Exhibit Potential Cooperativity in Membrane Binding

**Figure 6:**
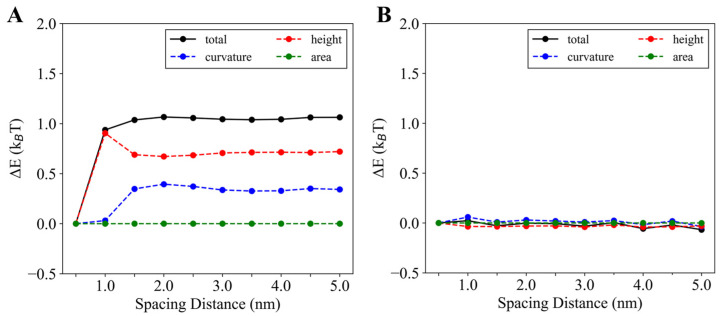
Membrane energy changes with the spacing distance for the parallel insertions (**A**) and series insertions (**B**). The membrane energy change ∆E is calculated as $∆E=E_{2}-E_{ref}$, where $E_{2}$ is the energy of the membrane with two helices inserted on the membrane and $E_{ref}$ is the energy of the reference membrane with an inter-helix distance of *d* = 0.5 nm. Here, simulations were performed with a DOPC membrane, and the helix parameters are *L* = 2 nm and $∆h_{0}$ = 0.15 nm.

The mixed membrane deformation induced by two helices suggests that the helices may exhibit some coupling effects or cooperativity during membrane binding. To assess this, we computed the membrane energy change (Δ*E*) due to two helix insertions, as shown in Figure 6. For parallel insertions, when the helices are far apart, ∆E remains unchanged with increasing inter-helical distance (Figure 6A). However, as the helices move closer together, (Δ*E*) decreases when the inter-helical distance is smaller than 1.5 nm. Analysis of the components of membrane energy revealed that as the inter-helical distance decreases from 5 nm to 0.5 nm, curvature energy starts to decrease from around 1.5 nm, while height energy increases slightly before decreasing drastically, with a peak at an inter-helical distance of 1 nm. In contrast, area elasticity shows negligible change. Thus, the fluctuation in (Δ*E*) is primarily driven by changes in membrane curvature and height induced by the helices. The decrease in (Δ*E*) as the helices come closer together suggests cooperativity between the two helices, as the system becomes more stable when the helices are closer due to a reduction in system energy. However, the total energy decrease of approximately 1 k_B_T for the inter-helix distance range of 5 nm to 0.5 nm is relatively small, indicating that the cooperativity is weak and unlikely to be experimentally observable.

We next examined ways to strengthen the cooperativity between the two helices. First, we ruled out helix arrangement, as our results show that series insertions do not exhibit significant changes in the energy-spacing curve (Figure 6B). This suggests that altering the helix alignment does not enhance cooperativity, and parallel insertions already exhibit the strongest cooperativity.

#### 3.2.4. Longer Helices and Deeper Insertions Enhance Cooperativity

**Figure 7:**
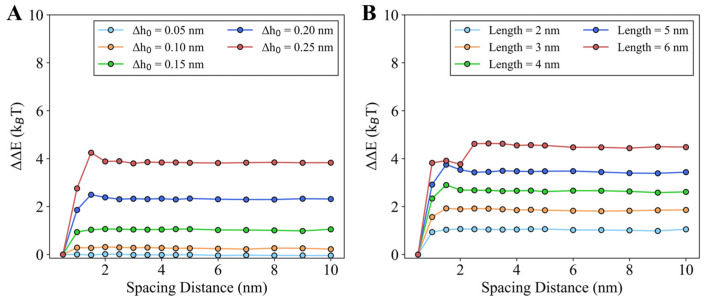
Energy-spacing relations are regulated by the helix length and helix insertion. (**A**) Effects of helix insertion depth, reflected by $∆h_{0}$, where the helix length is fixed as 2 nm. (**B**) Effects of the helix length *L*, where $∆h_{0}$ is fixed as 0.15 nm.

We further investigated the impact of helix length and insertion depth on the cooperativity of the two parallel insertions. By fixing the helix length at *L* = 2 nm, we found that deeper insertion or an increased $∆h_{0}$ value enhances the cooperativity of the two helices (Figure 7A). As $∆h_{0}$ increases, the energy-spacing curve shifts upward, leading to a larger reduction in membrane energy. This indicates that deeper insertions make the helices more likely to approach each other, further lowering the system energy. Notably, when $∆h_{0}$ exceeds 0.15 nm, the energy decrease due to helix clustering becomes significant (>2 k_B_T), making it experimentally detectable. Similarly, by fixing the insertion depth, $∆h_{0}$ = 0.15 nm, increasing the helix length also strengthens cooperativity. As shown in Figure 7B, longer helices cause the energy-spacing curve to elevate further, promoting clustering of the helices on the membrane. When the helix length exceeds 3 nm, the energy decrease due to clustering surpasses 2 k_B_T, which should be observable in experimental settings. Our simulation results suggest that both deeper insertion and longer helices enhance cooperativity in the two-helix system.

### 3.3. Multi-Helix Insertions Cooperate to Form Clusters on the Membrane

**Figure 8:**
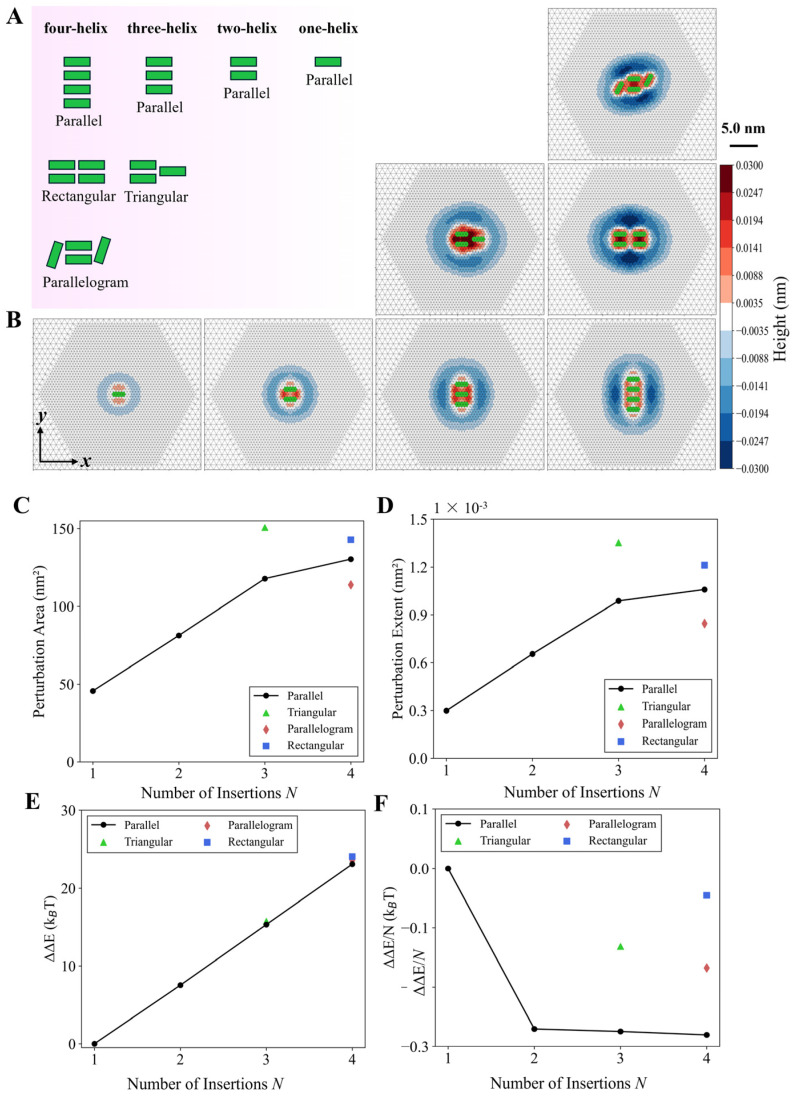
Membrane deformation induced by multi-helix insertions. (**A**) Schematics of helix arrangements for one-helix, two-helix, three-helix, and four-helix systems. (**B**) Mixed membrane deformation induced by multiple helices. The membrane is displaced in the *x*-*y* plane, with its normal along the *z*-axis, which is perpendicular to the paper and pointing outward. (**C**) The perturbation area (PA) varies with the helix number and arrangements. (**D**) The perturbation extent (PE) also varies with the helix number and arrangements. (**E**) Helix number and arrangement regulates the total membrane energy, and the energy per helix insertion (**F**). Simulations were conducted with the helix length *L* = 2 nm, $∆h_{0}$ = 0.2 nm, and the inter-helix distance is *d* = 1 nm.

Building on the robust evidence for the cooperativity between parallel helices through the membrane deformation, particularly at inter-helical distances < 5.0 nm and insertion depths ≥ 0.15 nm, we extended our investigation to explore more complex configurations. This extension was motivated by biological relevance, as cellular membranes typically contain diverse molecules that compete for interactions with phospholipids within confined spatial domains. As a result, we systematically studied the membrane deformation induced by varying both the number and spatial arrangement of helices. The number of helices was incrementally increased from 1 to 4, and their spatial configurations were modified accordingly. The configurations examined included parallel, triangular, rectangular, and parallelogram arrangements (Figure 8A). These arrangements represent tightly clustered helix insertions on the membrane.

As shown in Figure 8B, for the parallel alignments, several significant observations emerge as more helices are inserted on the membrane: (1) a progressive expansion of the membrane perturbation area and (2) an increased perturbation extent along the *z*-axis. These phenomena arise from the strengthened cooperative effects between multiple helix insertions. Further analysis of the perturbation area (PA) and perturbation extent (PE) profiles (Figure 8C,D) reveals several noteworthy trends. First, membrane deformation consistently increases with the number of helices, although this increase diminishes when the number of helices exceeds three. Second, membrane deformation depends on the helix arrangement. For example, the triangular configuration with three helices produces stronger membrane deformation than the parallel configuration in terms of both PA and PE. This enhancement is attributed to the stronger coupling between helices in the triangular arrangement, where every two helices strongly interact, while in the parallel arrangement, only adjacent helices experience significant coupling. In the four-helix system, both PA and PE exhibit a hierarchical order: rectangular > parallel > parallelogram arrangements. Similar to the three-helix triangular configuration, the rectangular arrangement in the four-helix system enables strong coupling between every two helices, resulting in the greatest membrane deformation among the three types of four-helix arrangements. The parallelogram arrangement yields the weakest membrane deformation, mainly due to its more complex geometry, where two inclined helices partially couple with the other two parallel helices at their ends, leading to unpredictable membrane perturbation.

The dependence of membrane deformation on multi-helix arrangement can be further substantiated by the system energetics, as shown in Figure 8E,F. For the parallel arrangements, the total energy of the system increases consistently with the number of helices (Figure 8E), while the energy per helix insertion decreases and then keeps almost unchanged when more than two helices are inserted into the membrane (Figure 8F), indicating a weakening of cooperativity among the helices. The energy profile in Figure 8F also suggests that as more parallel helices are inserted into the membrane, they tend to cluster closely together, reducing the average membrane perturbation caused by each helix. However, this clustering mechanism may not sustain all the insertions as one big cluster because the cooperativity among the helices is not stronger as the cluster size is bigger. Additionally, adjusting the helix arrangement does not result in larger helix cluster sizes, as other configurations tend to generate higher system energies compared to the parallel arrangement.

**Figure 9:**
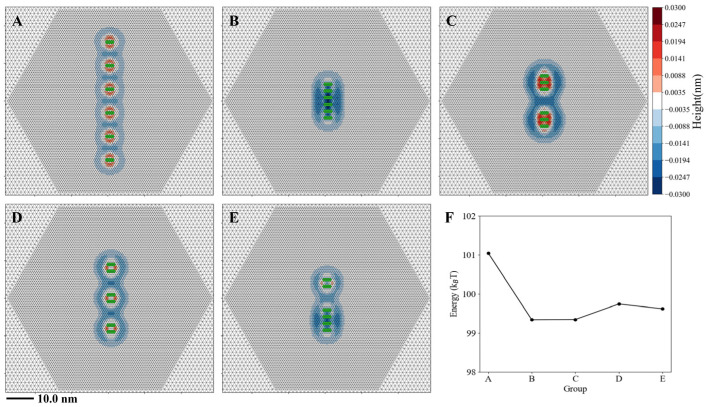
Six-helix systems tend to form clusters of varying sizes. (**A**) Six isolated helices with an inter-distance of 3.0 nm. (**B**) A single six-helix cluster. (**C**) Two three-helix clusters. (**D**) Three two-helix clusters. (**E**) One two-helix cluster and one four-helix cluster. (**F**) Energetics of Groups A, B, C, D, and E. All clusters maintain an inter-cluster distance of 3.0 nm and an inter-helix distance of 1.0 nm. Simulations were conducted using the DOPC membrane with the helix length of *L* = 2.0 nm and $∆h_{0}$ = 0.2 nm.

Since the helix cooperativity does not increase with the number of helices, this suggests that as more helices are inserted into the membrane, they may form clusters of varying sizes. To validate this inference, we conducted simulations with six parallel insertions arranged into five different distributions or groups (Figure 9). Group A represents six isolated insertions with an inter-distance of 3.0 nm, minimizing cooperativity in terms of energy. Group B consists of a single cluster of six helices with an inter-distance of 1.0 nm. Group C forms two clusters, each containing three helices, with a 3.0 nm distance between the two clusters’ edges, effectively isolating them. Group D consists of three isolated clusters, each with two helices. Group E features two clusters—one with two helices and the other with four. Our simulations reveal that although all five groups contain the same number of helices, they exhibit different energy levels (Figure 9F). Group A has the highest energy, indicating that helices tend to cluster rather than remain isolated. Groups B and C have the lowest energy, while Groups D and E show slightly higher energy than Group B. Notably, the energy differences among Groups B, C, D, and E are less than 1 k_B_T, making their differences negligible in the context of thermodynamics. Thus, from the energetic perspective, helices preferentially cluster on the membrane, but no single clustering pattern dominates. Instead, multiple cluster formations are equally likely. This further suggests that as more helices insert into the membrane, thermal fluctuations will make the formation of multiple, variably sized clusters rather than uniform arrangements.

## 4. Discussion

Shallowly inserted helices interact with biological membranes, inducing variable membrane deformations. We employ a continuum membrane model to quantitatively and qualitatively investigate the helix–membrane system. Our study demonstrates that even a single helix, with a relatively short length of 2 nm, can perturb the membrane anisotropically, while multiple helices lead to further notable phenomena.

To precisely quantify membrane deformation induced by helices, we calculate the membrane perturbation area (PA) and perturbation extent (PE), two terms that describe membrane deformation in a quantitative manner. It is important to note that PA and PE reflect structural changes in the membrane, but larger PA or PE values do not necessarily correspond to higher membrane energy. The membrane energy is defined based on the spontaneous curvature, according to the Helfrich–Canham–Evans functional. While PA and PE provide detailed insights into membrane deformation, including whether and how multiple helices interact or cooperate with the membrane, the membrane energy primarily indicates system stability. We propose that the combination of PA, PE, and energy offers a comprehensive and quantitative framework for describing the helix–membrane system from various perspectives.

Our analysis examined the relationships between the helix–membrane system by considering three key parameters: helix length, insertion depth, and inter-helix distance, capturing both helix varieties and their spatial arrangements. Despite exploring various conditions, our findings reveal consistent mechanistic principles governing membrane remodeling: (1) Helix length primarily modulates membrane deformation in both area and extent, whereas insertion depth mainly governs the extent of membrane deformation, underscoring the importance of helix properties; (2) The inter-helical distance plays a critical role in cooperative membrane deformation, with parallel insertions exhibiting a critical long-distance deformation, while other arrangements demonstrate short-distance deformation, reflecting the effects of helix distribution. Despite the influence of helix properties, the membrane properties also contribute to the helix–membrane interactions. Simulations with identical system setups but different membrane parameters, such as the DOPC vs. DLPC lipid membrane (Figure S5), show that while the quality of induced membrane deformations remains similar, the quantity varies. This highlights the significance of membrane properties and the necessity of using real biological parameter values to study helix–membrane interactions. These interconnected findings provide a unified framework for understanding the complex nature of helix-induced membrane deformation.

Our results indicate that the cooperative effect between helices can lead to their clustering of varying sizes. However, this phenomenon occurs only under specific conditions: helices must generate sufficiently strong perturbations in the membrane, either through their length or insertion depth. Our studies with biologically relevant parameter values suggest that short helices (*L* < 3.0 nm) cannot overcome thermodynamic barriers to exhibit strong cooperativity unless they have a large insertion depth ($∆h_{0}$ > 0.15 nm). In fact, in vitro experiments have not observed significant clustering of ENTH N-terminal helices (approximately 2 nm in length) [13], supporting our findings. Furthermore, other studies have reported strong helix cooperativity, referred to as membrane-mediated interactions between helices. These studies typically involve longer helices (e.g., 5 nm [36]) or assume that the helix can deform the membrane over an infinitely long distance along its axis [25], further corroborating our results. A recent experimental study observed α-synuclein multimerization on membranes [14]. α-Synuclein, like the ENTH N-terminal helix studied here, inserts shallowly into membranes. Our results here suggest that α-synuclein induces membrane deformation, leading to cooperative effects between nearby α-synucleins. The clustering is energetically favorable, consistent with experimental findings. Although our studies establish criteria for helix cooperativity, direct observation of helix clusters in experiments may require additional considerations, such as direct interactions between helices via electrostatic or other mechanisms, as well as the spatial resolution of experimental equipment, given that predicted helix clusters are small and local.

Using our continuum membrane model, we systematically investigate helix–membrane interactions. In addition to the robust findings presented above, our model allows for further exploration, thanks to its flexibility. For instance, helix arrangements could be considered on opposite sides of the bilayer membrane, accounting for biological membrane asymmetry, to simulate potential trans-membrane cooperation. Our membrane model assumes tight coupling between the two leaflets of the bilayer membrane. Including relative slip between the two leaflets and incorporating a more detailed description of helix–membrane interactions, like electrostatics and hydrodynamics, could provide additional insights into the roles of helix–membrane interactions in various biological processes.

## 5. Conclusions

We conducted a systematic study of shallowly inserted helices and their interactions with membranes using the continuum membrane model, capturing various aspects of membrane deformation. Our analysis reveals both the independent and coupled effects of membrane and helix properties, including helix length, insertion depth, and distribution. Our findings demonstrate that helix insertions exhibit a cooperative mechanism that regulates their binding to the membrane, and we provide criteria for evaluating helix cooperativity. This study offers valuable insights into the biological significance of membrane physical deformation induced by helices.

## Figures and Tables

**Table 1:** Parameters of the continuum membrane model and values for DOPC and DLPC membranes.

Parameter	DOPC	DLPC
Bilayer hydrocarbon height	2.71 nm [40]	2.09 nm [41]
Monolayer spontaneous curvature	−0.04 nm^−1^ [42]	+0.11 nm^−1^ [42]
Bilayer bending modulus	19.4 k_B_T [43]	20.4 k_B_T [43]
Bilayer area modulus	265 pN/nm [44]	234 pN/nm [45]
Monolayer height modulus	267 pN/nm [30]	165 pN/nm [30]

## Data Availability

Dataset available on request from the authors.

## Supplementary Materials

The following supporting information can be downloaded at: https://www.mdpi.com/article/10.3390/membranes15040101/s1. Figure S1. The membrane deformation of the outer layer and inner layer induced by a single helix with the length 1.0 nm and width 1.0 nm, or with the radius 1.0 nm. The resulting membrane deformation forms a circular shape; Figure S2. The membrane deformation of the inner layer induced by a single helix with varying lengths and insertion depths; Figure S3. Mixed membrane deformation changes with the spacing distance, depending on the insertion depth and helix arrangement; Figure S4. Two helices induce mixed membrane deformation of the inner layer. Figure S5. Helix-induced membrane deformation with DOPC (A) vs DLPC (B) compositions. Perturbation area and perturbation extent vary with the inter-helix spacing, where (C) and (D) are DOPC and DLPC membranes, respectively. Energy-spacing curve shows the helix cooperativity, (E) the left panel is the results for DOPC membrane, (F) while the right panel is for DLPC membrane. Simulations were performed with helix spontaneous curvature $c_{0,ins}=$ 0.3 nm^−1^, *L* = 2 nm, $∆h_{0}=$ 0.2 nm.
