# Supplementary material for: Membrane Remodeling Driven by Shallow Helix Insertions via a Cooperative Mechanism
Source: Membranes (Basel). 2025 Apr 1;15(4):101. doi: 10.3390/membranes15040101 (PMC12029183; doi:10.3390/membranes15040101)
Supplement: Supplementary file 1 [file membranes-15-00101-s001.zip › membranes-3437777-supplementary.pdf]

Supplementary Materials
for

Membrane Remodeling Driven by Shallow Helix Insertions via a Cooperative Mechanism

Jie Hu[1,2,3] , and Yiben Fu[3,4,*]

1. Key Laboratory of Biomedical Materials and Engineering of the Ministry of Education, South China University of Technology, Guangzhou 510006, P. R. China
2. National Engineering Research Center for Tissue Restoration and Reconstruction, South China University of Technology, Guangzhou 510006, P. R. China
3. School of Biomedical Sciences and Engineering, South China University of Technology, Guangzhou International Campus, Guangzhou 511442, P. R. China
4. Guangdong Provincial Key Laboratory of Biomedical Engineering, South China University of Technology, Guangzhou 510006, P. R. China

* Correspondence: yibenfu@scut.edu.cn

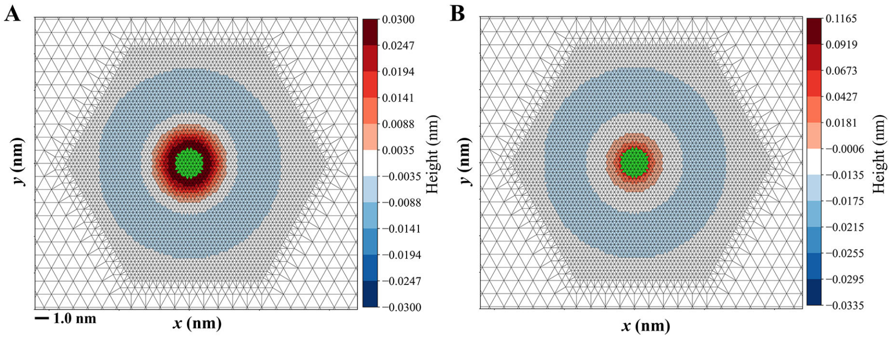

Figure S1. The membrane deformation of the outer layer (A) and inner layer (B) induced by a single helix with the length 1.0 nm and width 1.0 nm, or with the radius 1.0 nm. The resulting membrane deformation forms a circular shape. The simulation was performed with DOPC membrane, the spontaneous curvature at the insertion was $c_{0,ins}$= 0.2 nm^{-1} and Δh_0 = 0.15 nm.

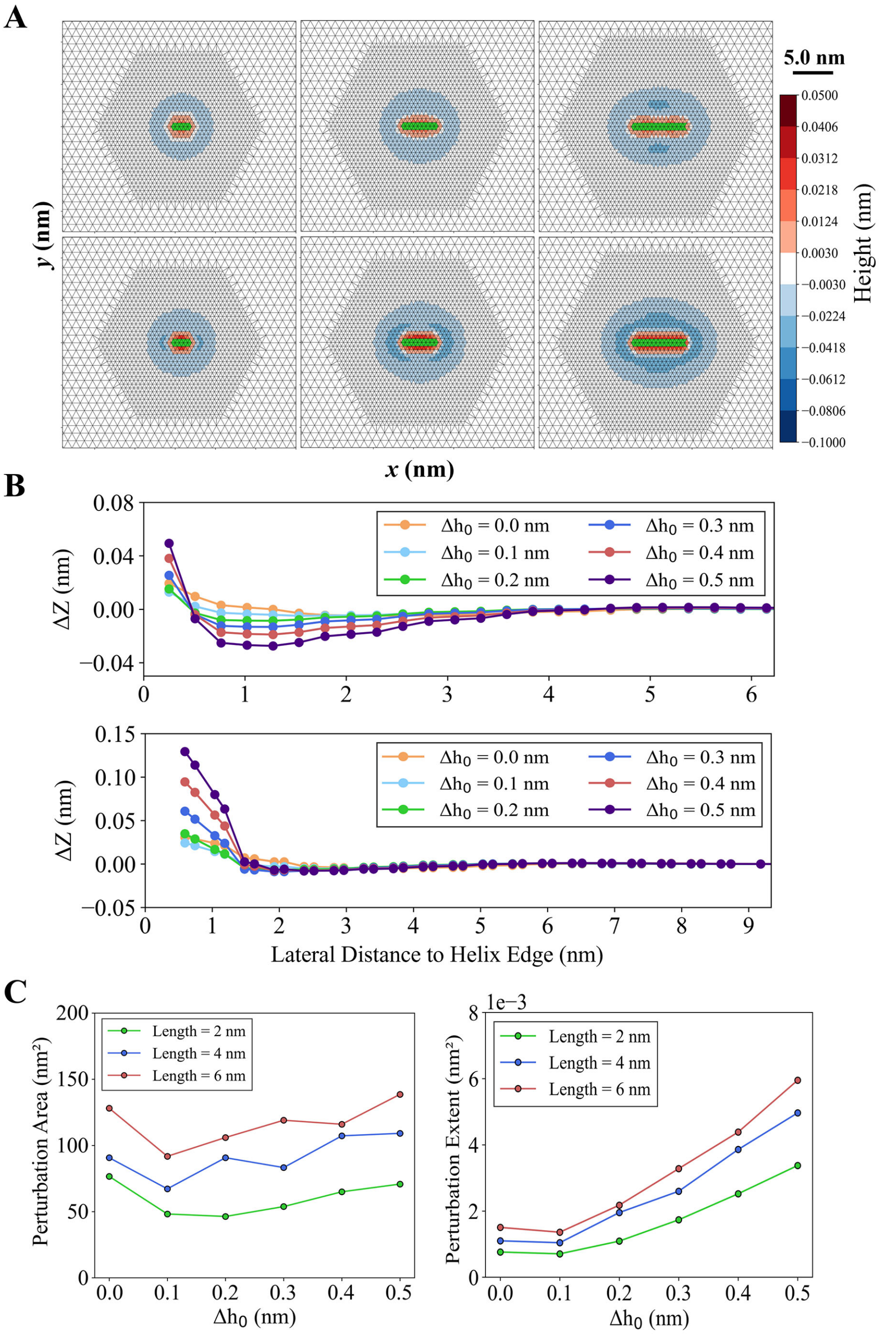

Figure S2. The membrane deformation of the inner layer induced by a single helix with varying lengths and insertion depths. (A) The three columns refer to three different helix length, from left to right, $L = 2$, 4, and 6 nm, respectively. The two rows, from top to bottom, refer to $\Delta h_0 = 0.1$ and 0.3 nm, respectively. Color coding depicts membrane thickness variations: red indicates increased thickness (elevation or arching), blue shows decreased thickness (thinning or sink), and green marks the location of the helix insertion. (B) Directional analysis of membrane height deformation around the helix insertion. The top figure represents the height profiles along the x-axis, and the bottom represents the height profiles along the y-axis. (C) Effects of the helix length and insertion depth on perturbation area and perturbation extent.

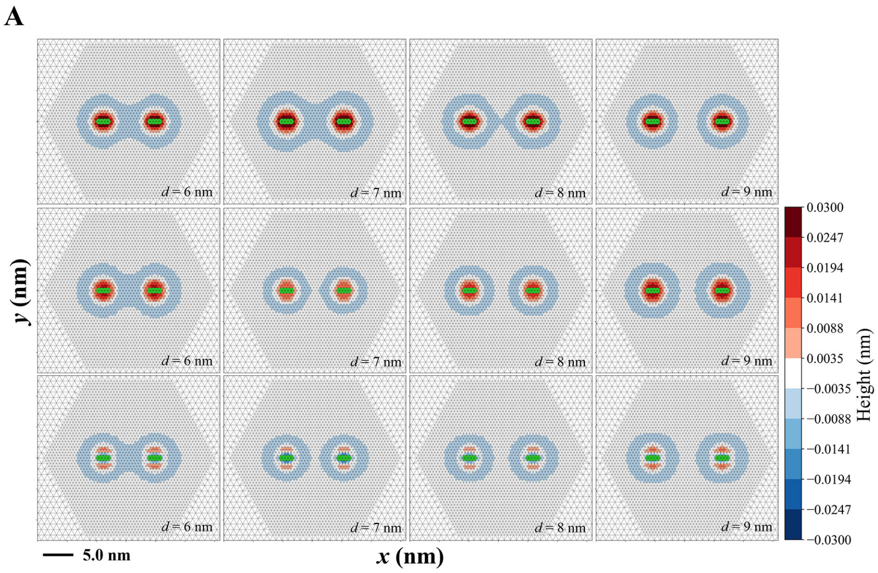

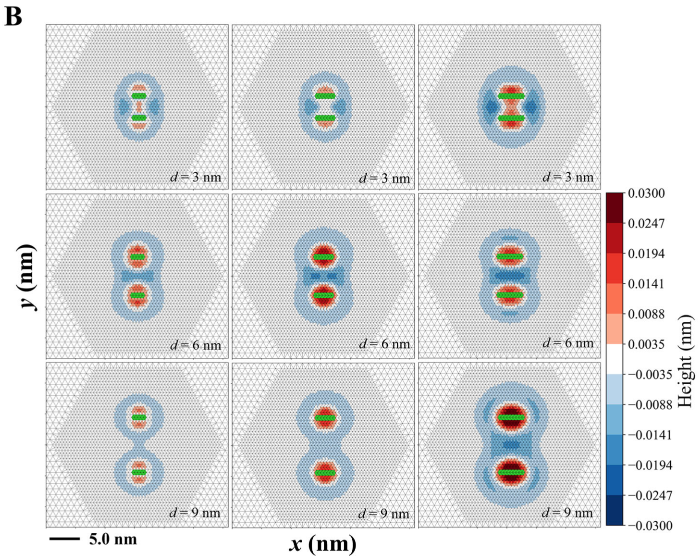

Figure S3. Mixed membrane deformation changes with the spacing distance, depending on the insertion depth and helix arrangement. (A) In the series insertion system, the merged area disappears at an inter-helical distance of about 7.0 nm. The four columns from left to right represent different inter-helix distance d = 6, 7 8, and 9 nm; the three rows from top to bottom represent Δh_0 = 0.05, 0.15, and 0.25 nm. (B) For parallel insertions, the mixed membrane deformation is stronger as the helix length is larger. From left to right, the helix lengths are 2, 3, and 4 nm, respectively.

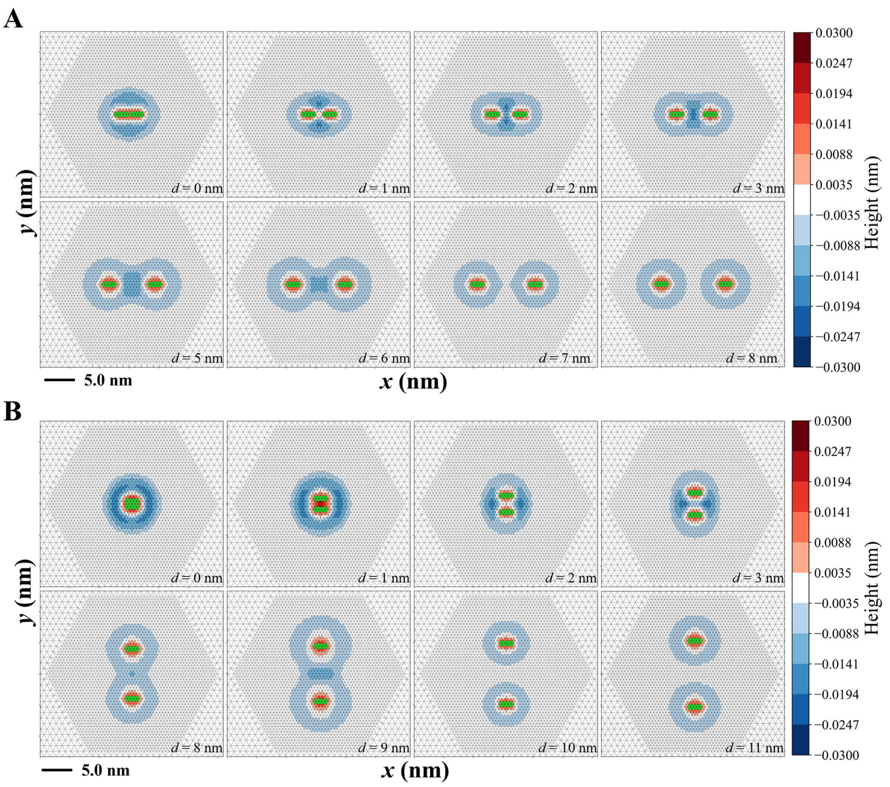

Figure S4. Two helices induce mixed membrane deformation of the inner layer. (A) Membrane deformation induced by series insertions. (B) Membrane deformation induced by parallel insertions. The helix length is 2 nm and Δh_0 = 0.15 nm.

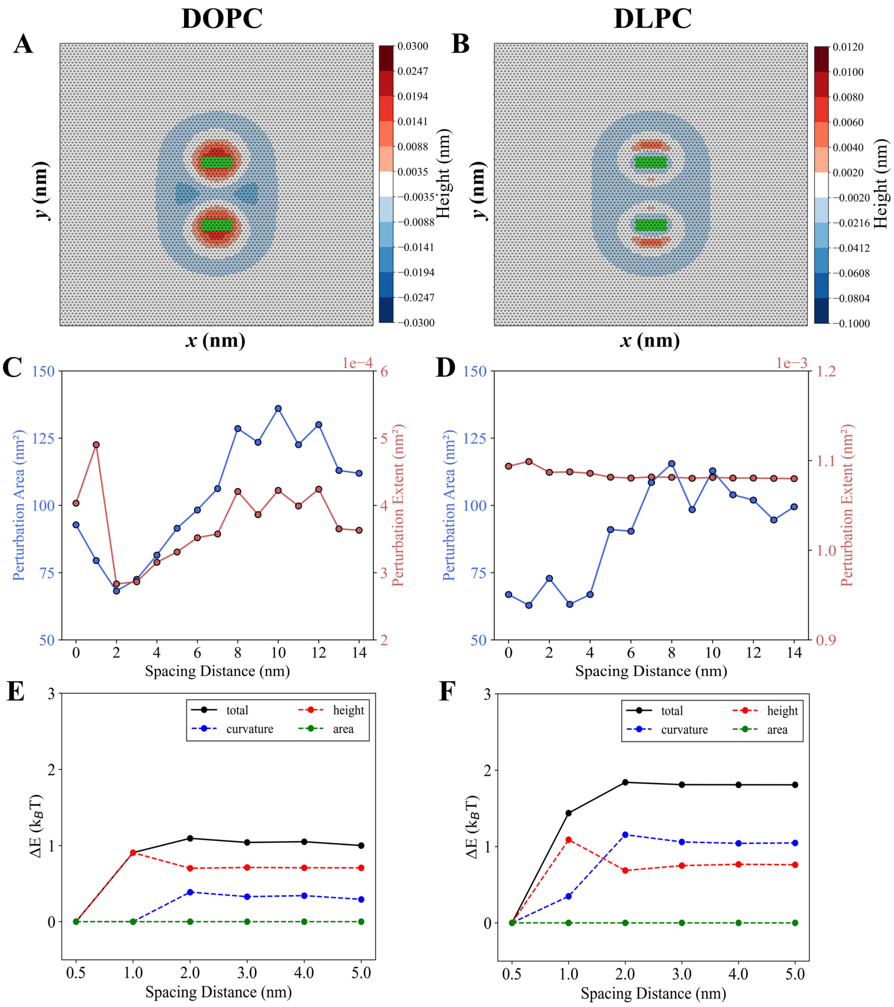

Figure S5. Helix-induced membrane deformation with DOPC (A) vs DLPC (B) compositions. Perturbation area and perturbation extent vary with the inter-helix spacing, where (C) and (D) are DOPC and DLPC membranes, respectively. Energy-spacing curve shows the helix cooperativity, (E) the left panel is the results for DOPC membrane, (F) while the right panel is for DLPC membrane. Simulations were performed with helix spontaneous curvature $c_{0,ins} = 0.3\ \mathrm{nm}^{-1}$, $L = 2$ nm, $\Delta h_0 = 0.15$ nm.
